# Is “Huh?” a Universal Word? Conversational Infrastructure and the Convergent Evolution of Linguistic Items

**DOI:** 10.1371/journal.pone.0078273

**Published:** 2013-11-08

**Authors:** Mark Dingemanse, Francisco Torreira, N. J. Enfield

**Affiliations:** 1 Language and Cognition Department, Max Planck Institute for Psycholinguistics, Nijmegen, The Netherlands; 2 Centre for Language Studies, Radboud University, Nijmegen, The Netherlands; Utrecht University, The Netherlands

## Abstract

A word like *Huh?*–used as a repair initiator when, for example, one has not clearly heard what someone just said– is found in roughly the same form and function in spoken languages across the globe. We investigate it in naturally occurring conversations in ten languages and present evidence and arguments for two distinct claims: that *Huh?* is universal, and that it is a word. In support of the first, we show that the similarities in form and function of this interjection across languages are much greater than expected by chance. In support of the second claim we show that it is a lexical, conventionalised form that has to be learnt, unlike grunts or emotional cries. We discuss possible reasons for the cross-linguistic similarity and propose an account in terms of convergent evolution. *Huh?* is a universal word not because it is innate but because it is shaped by selective pressures in an interactional environment that all languages share: that of other-initiated repair. Our proposal enhances evolutionary models of language change by suggesting that conversational infrastructure can drive the convergent cultural evolution of linguistic items.

## Introduction

A fundamental tenet of linguistic science is that the sound of a word has a purely arbitrary connection to the word's meaning [1], [2]. Thus, the sound of the word *dog* in English is connected to the concept ‘dog’ by historical accident and not by any natural connection; roughly the same concept is just as well denoted in French by *chien*, in German by *hund*, and in Japanese by *inu*. But it is not that a word can have just any vocal sound. While the possibility space for sound systems of the world's language is enormous, any given language makes use of only a restricted portion of the possible sounds [3], [4]. It follows from these two basic principles –the ‘arbitrariness of the sign’, and the ‘selectiveness of particular sound systems’– that the words that exist in the world's languages should sound quite different from each other, and that the likelihood that there are universal words is extremely small. But in this study we present a striking exception to this otherwise robust rule. From a systematic comparison of 10 spoken languages from 5 continents we find evidence suggesting that a word like ‘Huh?’–used as a ‘repair initiator’ when, for example, one has not clearly heard what someone just said [5], [6]–is a universal word.

There are two distinct claims being made here: 1. that *Huh?* is universal, and 2. that *Huh?* is a word. In support of the first claim, we show that the similarities in form and function of an interjection with the specific function of repair initiation are very much greater across languages than chance coincidence would admit. In fact the variation in form in unrelated languages across the globe is about the same as the variation we find in the way any regular word (e.g., *dog*) is pronounced across dialects of English. In support of the second claim, we show that *Huh?* meets the criteria of a word in the sense of being a conventional lexical sign which must be learnt. Thus, in contrast to what has been presumed for interjections in general [7], [8] and for *huh?* in particular [9], [10], we find that this item is linguistic in nature rather than being a mere grunt or non-lexical sound. We show that the form is locally calibrated in ways that show it fitting within different language systems. *Huh?* may be a non-prototypical word, but it is a word.

Finally, we address the question of why all languages should have such a word and why its form should be so similar across languages. We observe that this item fulfils a crucial need shared by all languages –the efficient signalling of problems of hearing and understanding– and we propose that its form is constrained by selective pressures in a conversational environment that is essentially the same in all languages. Consider a case from English [10]:

**Extract 1** American English [NB, 1:1:19]



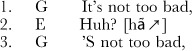



After speaker G makes a statement, speaker E utters the interjection *huh?*. This is followed by a repetition of the original statement by G. The technical term for this type of sequence is “open other-initiated repair”: repair is initiated not by the speaker of the first turn but by the other participant (“other-initiated”), and the repair initiator signals that there is a problem, but it leaves open what the problem is (“open”) [11]. The actual repair operation in response to this interjection is usually simply repetition, sometimes with slight modification. Extracts 2 and 3 show structurally identical sequences in two other languages: Siwu, a Kwa language spoken in Ghana, and Lao, a Tai-Kadai language spoken in Laos.

**Extract 2** Siwu (Ghana) [Maize1_1017013]



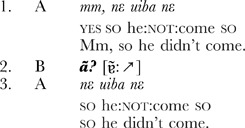



**Extract 3** Lao (Laos) [CONV_050815c_03.10]



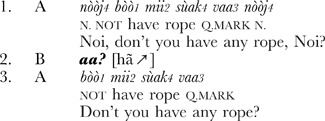



These examples show that it is possible to identify the same conversational structure in unrelated languages. Essentially, this method gives us a *natural control* over conversational data, making possible systematic comparison across languages [12], [13]. Sequences of other-initiated repair have been identified in every spoken language investigated so far [14], [15], and as the examples show, the interjection in the pivotal turn can be remarkably similar. This leads to the question driving our study: is *huh?* in this context a universal word?

**Figure 1:**
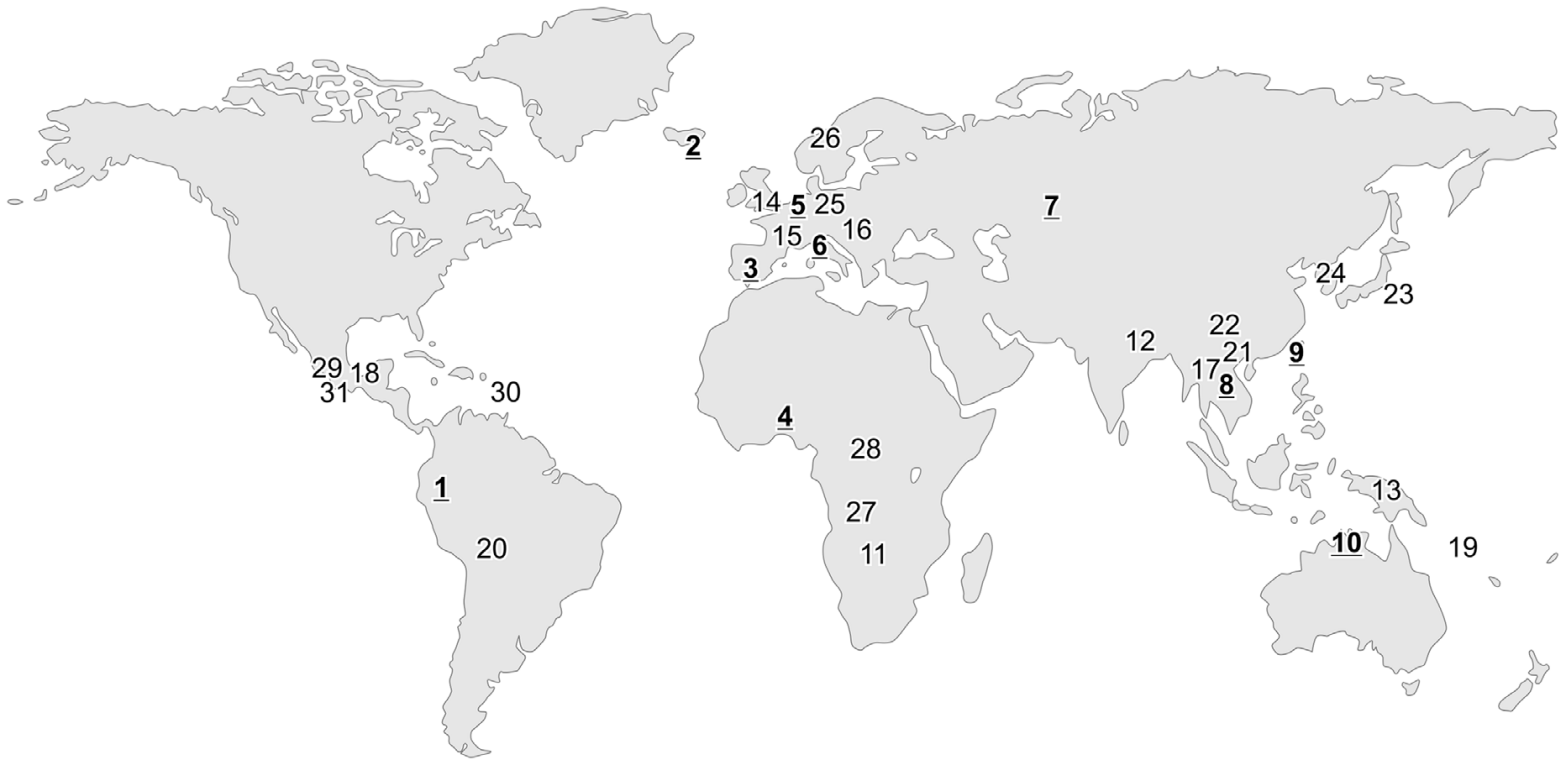
Interjections for other-initiation of repair in thirty-one languages. A word like *huh?* –used to initiate repair when, for example, one has not clearly heard what someone just said– is found in roughly the same form in spoken languages across the globe. Languages 1–10 are examined in detail in the present study, 11–20 from [14], 21–31 from sources cited. Locations are approximate. 1. *Cha*'*palaa* 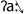 2. *Icelandic* ha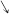 3. *Spanish* e↗ 4. *Siwu* ã:↗ 5. *Dutch* h
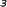
↗ 6. *Italian* ε:↗ 7. *Russian* a:↗ 8. *Lao* hã:↗ 9. *Mandarin Chinese* ã:↗ 10. *Murrinh-Patha* a:↗ 11. *‡Âkhoe Hai//om* hε↗ 12. *Chintang* hã↗ 13. *Duna* 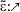 14. *English* hã↗ 15. *French* 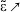 16. *Hungarian* hm↗/ha↗ 17. *Kri* ha:↗ 18. *Tzeltal* hai↗ 19. *Yélî Dnye* 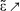 20. *Yurakaré* æ↗ 21. *Lahu* hãi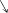 [38] 22. *Tai/Lue* hy ˘↗/há↗ [92] 23. *Japanese* e↗ [93] 24. *Korean* e↗ [94] 25. *German* h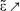 [95] 26. *Norwegian* h
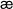
↗ [96] 27. *Herero* e↗ [97] 28. *Kikongo* e↗ [98] 29. *Tzotzil* e↗ [99] 30. *Bequia Creole* ha:↗ [100] 31. *Zapotec* aj↗ [101].

By compiling data from published literature we found that in thirty-one languages around the world, the interjection for other-initiated repair appears to be strongly similar (Figure 1). However, written sources are rarely explicit about the precise form, meaning, and use of interjections. The most reliable way to study a conversational interjection is by examining cases of actual use. Therefore we collected data from recordings of naturally occurring informal conversations in a sample of 10 languages from 5 continents, varying fundamentally in terms of phonology, word structure, and grammar (languages 1–10 in Figure 1). For optimal comparability, we studied the exact same conversational environment across languages: that of other-initiated repair (OIR), in which one participant produces a turn at talk, the other then signals some trouble with this turn, and finally the first produces a next turn which aims to solve the trouble, usually by means of repetition and/or modification. In some languages the interjection, or an item similar to it, was also found in other sequential environments, for instance to mark surprise or to pursue a response. Such alternative (and probably derived) uses provide insight in possible paths of semantic change, but we exclude them here to make sure we are comparing like with like.

Earlier we found that all 10 languages in the sample make available two types of expressions to initiate repair in this conversational environment [14]: an interjection (comparable to English “huh?”) and a question word-based expression (comparable to English “what?”) – with the interjection being a dedicated, default form for open other-initiated repair, and the question word being recruited from a larger grammatical paradigm of question words. The question words for initiating repair in the languages in our sample are very different in phonetic form, with varying numbers of syllables, a wide range of different consonants and vowels, and many different combinations of speech sounds (Table 1). This is just as expected in a diverse language sample given the principle of the arbitrariness of the sign. Compared to the question words, the interjections for initiating repair are strikingly similar in form (Table 1). It is this exceptional similarity that we investigated in this study.

**Table 1:** Question words (“what?”) and interjections (“huh?”) for initiating repair in ten languages.

Language	Question word	Interjection
Cha'palaa	ti	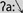
Dutch	wat	hɜ↗
Icelandic	k^h^va ː θ	ha 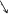
Italian	k^h^ɔza	εː↗
Lao	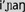	hãː↗
Mandarin Chn.	ʂəmə	ãː↗
Murriny Patha	taŋgu	ãː↗
Russian	ʃtɔ	aː↗
Siwu	beː	ã:↗
Spanish	ke	e↗

## Materials and Methods

We collected 196 instances of the interjection for other-initiated repair (henceforth OIR interjection) in videotaped recordings of conversation in a worldwide sample of 10 languages (mean instances per language  = 19.6, sd  = 7.5). We used field recordings of maximally informal conversation because most written sources do not offer enough phonetic detail and people's intuitions about their behaviour can be different from their actual behaviour [16]. We examined at least ten tokens per language to find out whether or not the same articulatory target is aimed for within and across languages. All data were collected in accordance with protocols approved by the ethical review board of the Seventh EU Framework (240853 HSSLU). Informed consent was obtained from all participants according to standard practices [17], [18]. The data were anonymised and unlinked and there is no possibility of identification.

We used a two-stage approach to comparative analysis of the tokens. In an auditory analysis, we collected phonetic auditory judgements of interjection tokens by three annotators and combined them into graded measures along five phonetic dimensions for every single token (see File S1). In an instrumental analysis, we took acoustic measurements on a subset of tokens and used these to verify the auditory judgements for selected dimensions. The combination of auditory and instrumental approaches enabled us to carry out an analysis that is ecologically valid and well controlled.

In the auditory analysis, all interjection tokens (n = 196) were presented one by one in random order to three annotators independently. No separate information about language or recording was provided. Annotators listened to the audio clips with spectrograms and pitch tracks available on screen, and coded every token for five phonetic dimensions selected on the basis of preliminary observations of the range of variation: closure, nasality, vowel quality, intonation, and onset (see SI). Articulatory gestures in spoken language are essentially gradient [19]. Therefore, the coding results were combined into cumulative measures per token per dimension, allowing us to measure and display the variation in, for instance, vowel quality or consonant onset by language.

In the instrumental analysis, we took acoustic measurements of intonation and the first two vowel formants for languages in which token quantity and acoustic quality permitted this, namely Spanish and Cha'palaa. For Spanish, all tokens came from laboratory recordings of casual conversation [20]; for Cha'palaa, the large number of tokens in the field recordings permitted instrumental analysis. Some acoustically inferior interjection tokens (due to overlapping speech or ambient noise) and some tokens spoken by children were discarded. In total, 13 Cha'palaa tokens and 12 Spanish tokens were analysed instrumentally. Pitch values throughout each interjection were computed, and formant values of vowels were measured at the point of maximum intensity using the Burg method implemented in the software Praat [21].

## Results

All interjection tokens in all languages in our sample are syllable-like utterances consisting of one vowel-like sound optionally preceded by a consonant-like sound. We refer to these elements as syllable, vowel, and onset. We never found forms longer than one syllable and we never found any final consonant-like sounds.

### Vowels

**Figure 2:**
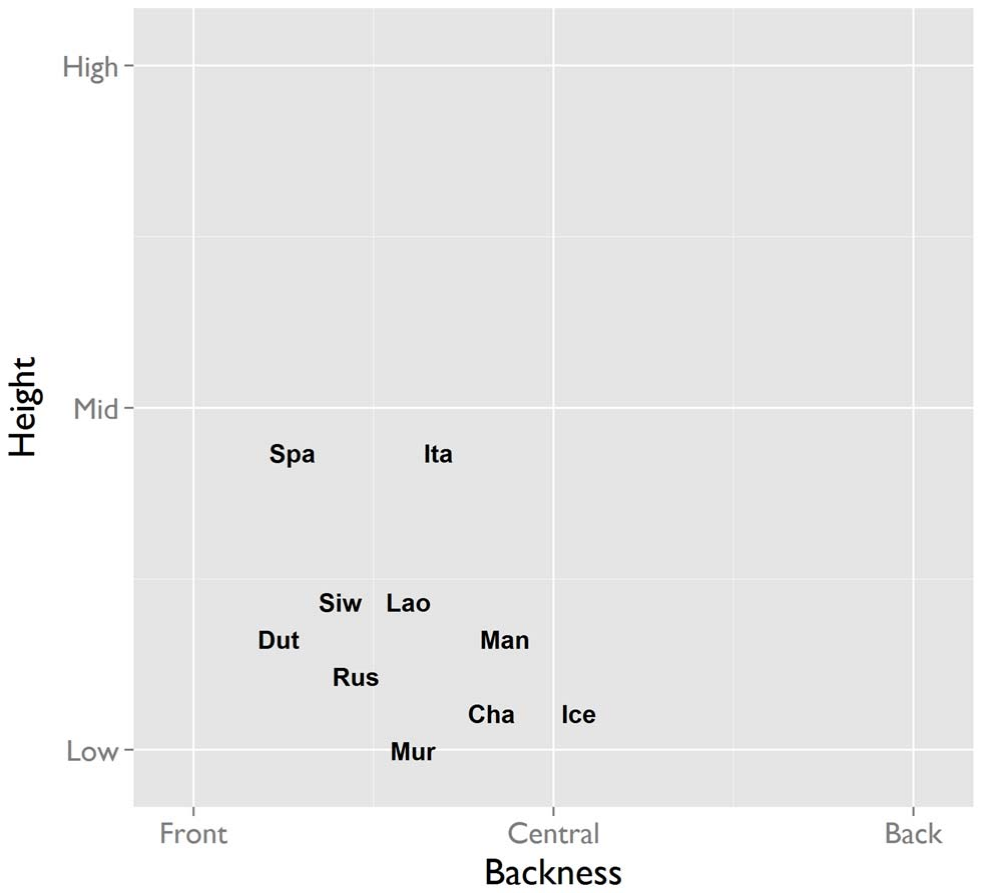
Average positions of the interjections in vowel space. The vowel inventories of the world's languages tend to make maximal use of vowel space [41]. In contrast to this, the vowels of the OIR interjections all cluster in the same low-front region. Abbreviations: Cha'palaa (Cha), Dutch (Dut), Icelandic (Ice), Italian (Ita), Lao (Lao), Mandarin (Man), Murrinh-Patha (Mur), Russian (Rus), Siwu (Siw), Spanish (Spa).

**Figure 3:**
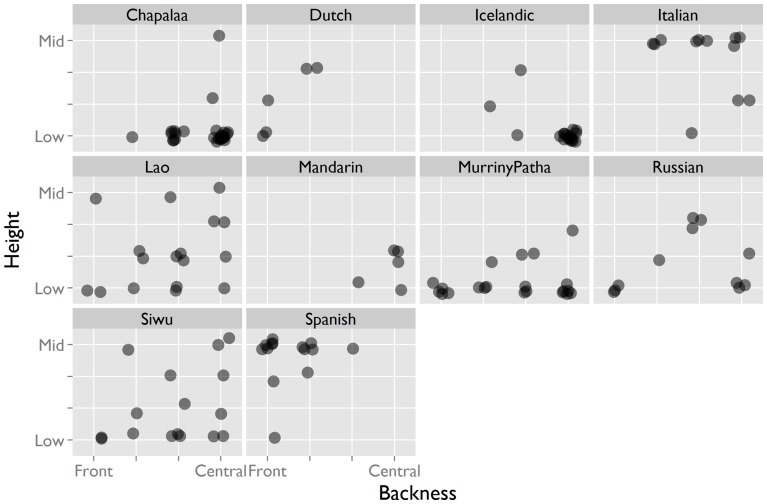
Vowel quality of interjection tokens by language. Although the vowel of the OIR interjections is limited to the low-front region, auditory analysis shows that within that region, not all languages target the same spot – the interjections appear to have distinct vowel targets.

Vowels can be characterized along three continuous articulatory dimensions: height (referring to the height of the tongue, associated with the F1 formant), backness (referring to the position of the tongue relative to the back of the mouth, associated with the F2 formant), and lip rounding. Within the two-dimensional space formed by height and backness, OIR interjections occupy only the low front central corner (Figure 2). Coding divided this corner of the space into four perceptual quadrants along two dimensions: Height (from low to mid) and Backness (from front to central). Within this restricted part of the space, most languages appear to aim for specific local targets (Figure 3). For instance, Cha'palaa tokens cluster in the low-central region, Spanish has a preference for the mid-front region, Italian clusters in the mid-central region, and Murrinh-Patha is mostly low. Some languages have a wider spread than others (e.g. Lao, Siwu). With respect to the third dimension, that of lip rounding, we found no variation: only unrounded vowels were found in all of the languages.

**Figure 4:**
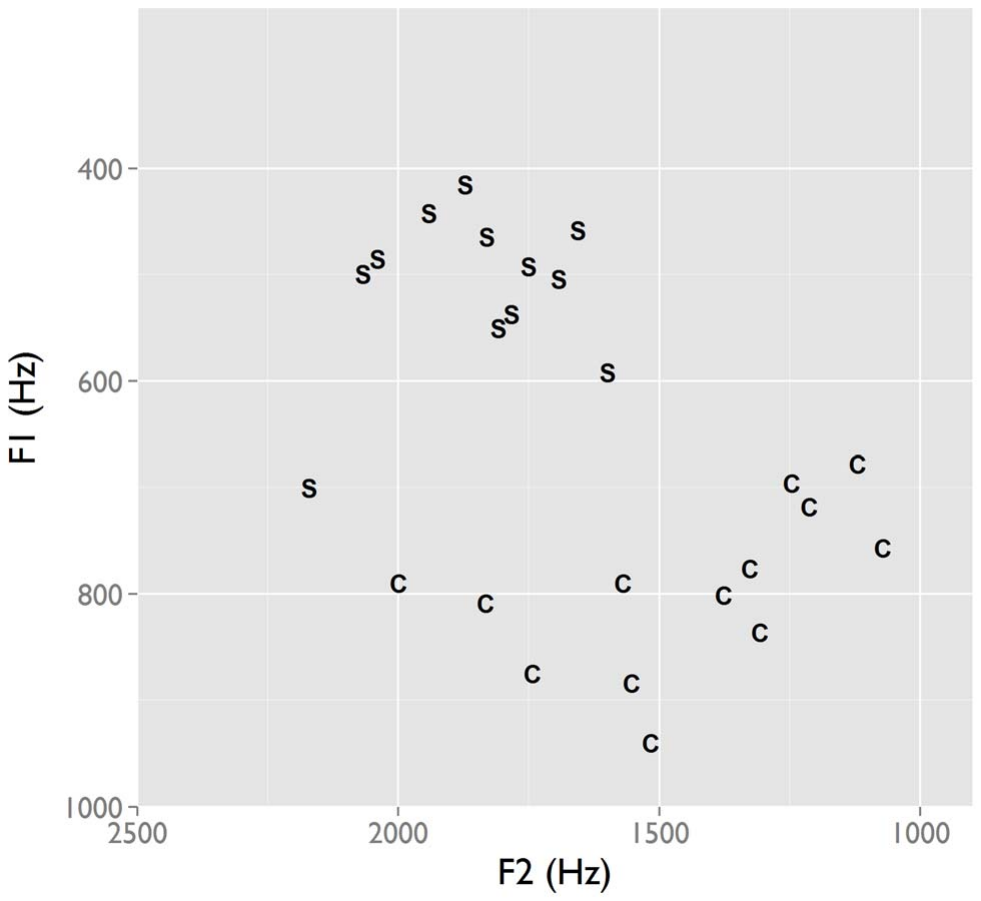
Formant values for the interjection vowels in Spanish (S) and Cha'palaa (C). An instrumental analysis of interjection tokens from Spanish and Cha'palaa shows that the interjections have distinct, language-specific vowel targets, confirming the auditory analysis in Figure 3.

To verify the validity of the auditory judgements, we performed acoustic measurements of the first two vowel formants (F1 and F2, associated with the height and backness dimensions) for Spanish and Cha'palaa (Figure 4). The two languages are statistically different on both of these dimensions (F1: B = −284.65, t = −9.34, p<.0001; F2: B = 398.54, t = 4.2, p<.0001). Spanish tokens have lower F1 and higher F2 values, consistent with the mid front vowel [e] found in the auditory judgments. The F1 of the Spanish interjection is in the same range as the F1 of the /e/ in a corpus of spontaneous Spanish [22], making it likely that the articulatory target of the interjection fits the phonology of the language. Cha'palaa tokens have higher F1 and lower F2 values, consistent with the low central vowel [a] found in the auditory judgements.

### Intonation

**Figure 5:**
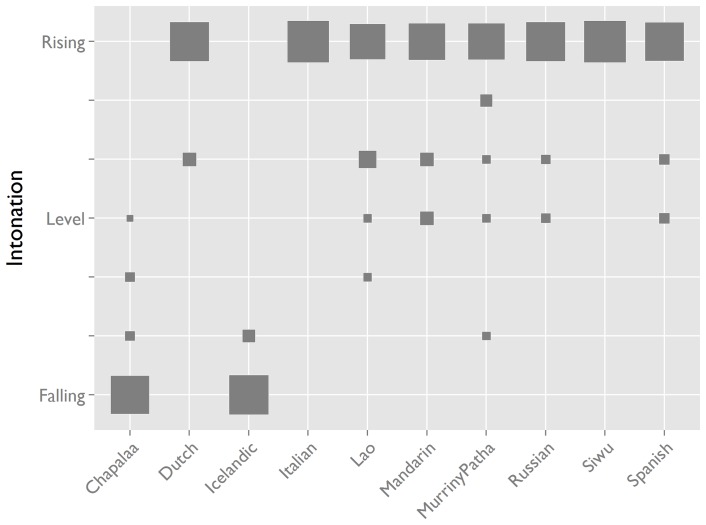
Intonation of the OIR interjection by language. Intonation of the OIR interjection is rising in most languages and falling in some, but more accurately described as “questioning” in all. In this product plot [102], area of squares is proportional to token count: a larger square means more tokens.

Within languages, the intonation of the interjection tokens is strongly consistent. In most languages in our sample it has rising pitch (Figure 5). Across many languages, rising pitch is associated with non-finality, uncertainty, and questioning [23], [24]. Exceptionally, in two languages in our sample the interjection has falling pitch: Icelandic and Cha'palaa. In these languages, falling intonation is the preferred intonation in wh-questions [14], [25], and the interjection shares its intonation with the question word-based expression for open repair initiation. The falling pitch of the OIR interjections in these languages thus appears to be calibrated to the local system of interrogative prosody. Across languages, then, the pitch of the interjections is best described as “questioning”, with the specific formal value determined by the local language system.

**Figure 6:**
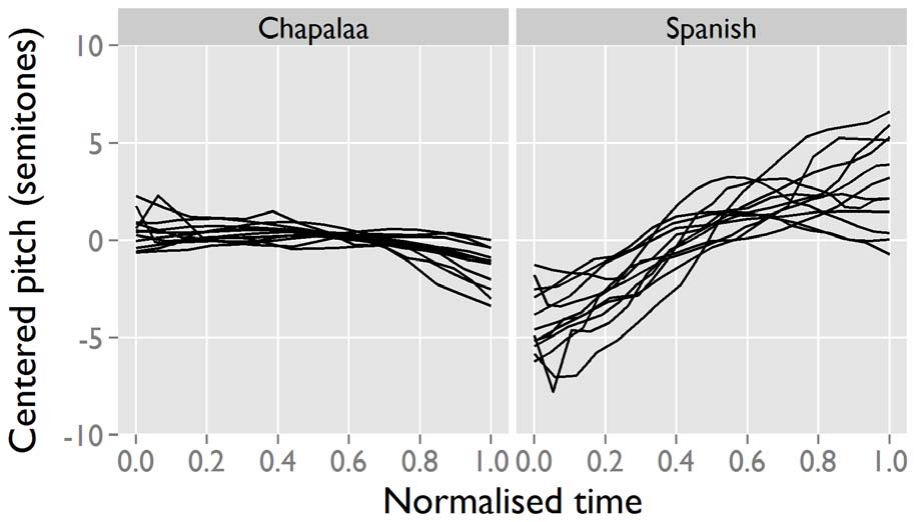
Pitch tracks for interjections in Spanish and Cha'palaa. Instrumental analysis of pitch tracks in Spanish (n = 12) and Cha'palaa (n = 13) confirms the auditory analysis in Figure 5.

To verify the validity of the auditory judgements we performed acoustic measurements on Spanish and Cha'palaa interjections, calculating pitch excursion as the difference between the pitch at the beginning and end of the contour. Pitch tracks in normalised time show rising contours for Spanish and falling contours for Cha'palaa, consistent with the auditory judgements (Figure 6). Spanish contours rise around 7 semitones on average, while Cha'palaa fall around 2 semitones. A model with pitch excursion as dependent response and language as predictor shows that the difference between the groups is statistically significant (B = 9.01, t = 8.04, p<.0001).

### Onset

**Figure 7:**
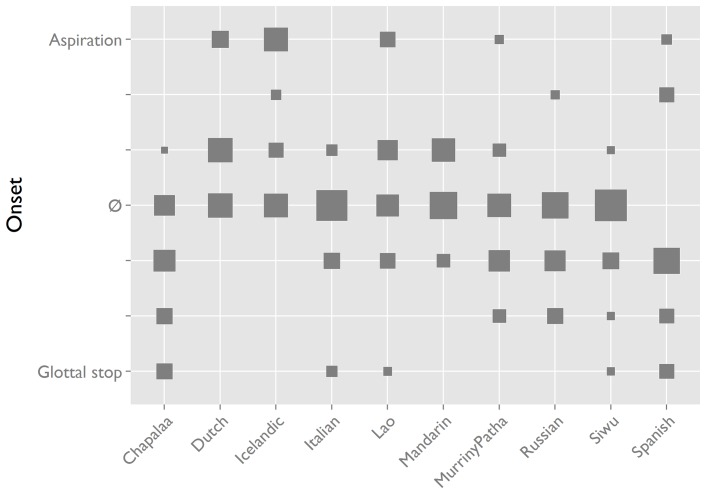
Interjection onset by language. *Aspiration* [h] and *glottal stop* [ʔ] onsets are at opposite ends of a continuum with *no onset* (*
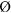
*) in the middle. In most languages, ‘no onset’ is the default form, and the direction in which interjections diverge from this is related to the phonology of the language. Spanish is a special case because the laboratory recordings [20] allow the detection of even the slightest glottal constriction or aspiration. In this product plot [102], area of squares is proportional to token count: a larger square means more tokens.

Most interjection tokens in most languages have no onset, but if there is one it is restricted to a glottal stop [ʔ] or a glottal fricative [h] (Figure 7). The direction in which tokens diverge from the no onset default appears to be influenced by the phonological system of the language, as follows: *if* an interjection token features an onset, that onset tends to approach one of the glottal consonants found in the phoneme inventory of the language.

Thus, Cha'palaa has a phonemic glottal stop/ʔ/[26], and many of its interjection tokens feature this sound. Dutch and Icelandic have phonemic/h/in onset position [27]–[29] and many of their interjection tokens feature this sound. Lao has both/ʔ/and/h/as distinctive sounds in onset position [30] and the onsets of its OIR interjection vary between /ʔ/,/ h/, and zero. The reverse holds true as well: if there are no glottal sounds in the phonology of the language, there is a high probability that the interjection will not feature a clear onset consonant. Thus, Mandarin, Murrinh-Patha and Russian have no phonemically contrastive glottal stop or fricative [31]–[33], and none (Mandarin & Russian) or very few (Murrinh-Patha) of their OIR interjection tokens feature these sounds. As Figure 7 also shows, the relation between the consonant inventory of the language and the onset of the interjection is not deterministic: presence of glottal consonants in the phonological inventory does not predict the occurrence of a consonant in all of its OIR interjections.

**Table 2:** Languages, field sites, and contributing researchers.

Language	Phylum	Field site	Researcher
Cha'palaa	Barbacoan	Ecuador	Simeon Floyd
Dutch	Germanic	The Netherlands	Mark Dingemanse
Icelandic	Germanic	Iceland	Rósa Gísladóttir
Italian	Romance	Italy	Giovanni Rossi
Lao	Tai	Laos	Nick Enfield
Mandarin Chn.	Sinitic	Taiwan	Kobin Kendrick
Murrinh-Patha	Southern Daly	Northern Australia	Joe Blythe
Russian	Slavic	Russia	Julija Baranova
Siwu	Kwa	Ghana	Mark Dingemanse
Spanish	Romance	Spain	Francisco Torreira

### Nasality and mouth aperture

Some degree of nasality of the vowel was perceived in the majority of cases (Figure A in File S1). This may be connected to the fact that a slightly lowered velum is the neutral or resting position for the articulators [34]. It may also be linked to the glottal quality of the consonant onset; the affinity between nasality and glottality is well-known and has been explained on perceptual as well as articulatory grounds [35], [36].

Closed-mouth variants of the OIR interjection (e.g. *m? n?*) were observed in most of the languages in our sample, but the overall frequency was low and it was not the most common form in any of the languages (Figure B in File S1). Qualitative analysis suggests that closed-mouth forms are mainly used when speakers are close to each other. Since bilabial closure and a lowered velum are the neutral position of the speech articulators [34], these closed forms may be seen as an extreme articulatory reduction of the open-mouth form.

## Discussion

### Is *huh?* a word?

In work on English conversations, the interjection *huh?* has been characterised as a “non-lexical token” [9] or a “non-lexical conversational sound” [8]. Yet our phonetic analysis shows that despite the overall similarity across languages, the OIR interjection is systematically calibrated to the language system in which it is integrated. This motivates the question whether *huh?* is a word. Two key characteristics of words are ‘integration’ – they are items in larger linguistic systems, and ‘conventionalisation’ – one cannot know them without having learnt them. Non-linguistic vocalisations like crying or grunting are the opposite on both counts: they are not integrated in linguistic systems, and one does not need to learn them to know them.

#### Integration

In all languages investigated, the sound of the OIR interjection shows some degree of calibration to local linguistic systems. Vowel targets are language-specific and appear to be drawn to existing phonemic targets, e.g. /e/ for Spanish and /a/ for Cha'palaa. Intonation melodies appear to be linked to the interrogative prosodic system, which may differ from language to language. The occurrence and quality of consonant onsets is related to the consonant inventory of the language. The interjection is also part of a larger paradigm of expressions for the other-initiation of repair, including, in English, other items like *what?* and *pardon?* [5], [15]. *Huh?* is thus an item integrated in several linguistic subsystems, from segmental and prosodic phonology to conversational structure.

#### Conventionalisation

*Huh?* exhibits linguistic conventions that speakers need to learn in order to use the form properly. A learner of Spanish has to know that repair is initiated with the mid front unrounded vowel “e↗”, a learner of Cha'palaa has to know that the form is more like “a
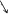
” with falling intonation, and a learner of Dutch has to know that a glottal fricative at onset is common: “h
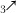
”. Its acquisition follows a normal trajectory, at least in American English-speaking children [37]. Second language learners' reports confirm that the precise form of this interjection has to be learnt, and that intuitions are not necessarily a reliable guide in this process [38].

Perhaps there is a continuum from non-linguistic vocalisations like sneezing and crying to prototypical conventional lexical items like *bless you* and *pain* [39]. Our evidence suggests that *huh?* is more on the word side of that continuum. Based on the fact that *huh?* is integrated in multiple linguistic subsystems and conventionalised in language-specific ways we conclude that *huh?* a lexical word.

### Is *huh?* universal?

Although there is systematic calibration to specific language systems, the bandwidth of the variation of OIR interjections across languages is exceedingly narrow. In all languages investigated, it is *a monosyllable with at most a glottal onset consonant, an unrounded low front central vowel, and questioning intonation*.

#### Narrow bandwidth of variation

We have already shown that the uniformity of the interjections is in striking contrast to the question words that languages can recruit for the same function (Table 1). Another way to appreciate the small range of cross-linguistic variation exhibited by this form is to consider it in context of the possibility space for words in spoken languages. Across languages, words can consist of one or more syllables, but the OIR interjection was never longer than one syllable in the languages we have studied, even in those like Murrinh-Patha, for which phonological words are generally longer than one syllable. Across languages, syllables can have rich internal structure, but the only structure attested in the OIR interjection is (C) V, i.e. a vowel V with an optional onset consonant C, even in languages like Dutch, where CVC syllables are common.

#### Similarity in vowels and consonants

Strong constraints on variation are also seen in the vowels and consonants employed. Vowel space can be depicted as a two-dimensional plane formed by height and backness. On average, languages have around 6 vowel phonemes [40], which tend to be maximally spread across this space to increase perceptual distinctiveness [41]. Given this fact, it is striking that the vowels of OIR interjection tokens are only found in the low front central corner of vowel space (Figure 2), and that on a third dimension of lip rounding OIR interjections are only found on the ‘unrounded’ side. Consonants are articulated at different locations throughout the vocal tract (lips, teeth, alveolar ridge, palate, velum, uvula, pharynx, epiglottis) and with different manners of articulation, from plosives, nasals and trills to taps, fricatives, and glides – a multidimensional possibility space in which the International Phonetic Alphabet records at least 64 simple phonemic consonants (and three times as many complex variants) attested in the world's languages [4]. Out of this enormous range of possibilities, only *two* basic sounds, the glottal consonants [ʔ] and [h], are found in the OIR interjection across languages.

Such limited variation and striking similarity across languages is wholly unexpected on the basis of the principle of the arbitrariness of the sign. Does this mean that *huh?* is a universal word? We propose a qualified yes. Qualified, because *huh?* is clearly not phonetically the same word across languages – if Cha'palaa tokens were cross-spliced into Spanish dialog, Spanish speakers would likely be confused. What appears to be universal is the function of this interjection along with a set of constraints determining its form. Other-initiated repair sequences have been found in all languages investigated so far, and no language appears to lack an interjection for this function. Thus *huh?* is universal in the sense that a short, questioning interjection like it with the function of initiating repair is likely to be attested in all natural spoken languages.

### Possible explanations

As we have seen, *huh?* is so common as to be practically universal, and yet calibrated to specific language systems such that it qualifies as a word. The language-specific nature of words is of course expected; it is the strong similarity that is in need of an explanation. Why do we find basically the same form –something like *huh?*– everywhere and not, say, *bi* in one language and *rororo* in the next? We consider two possible explanations. The first is that *huh?* is similar across languages because it is an innate grunt. The second is that it is similar as a result of convergent evolution. Empirical evidence supports the second.

#### Innateness

On one account, *huh?* may be similar across languages because it is a natural symptom with a biological basis, like laughs and screams – a “grunt” [8], [10]. Such qualifications, common in the wider literature on interjections, place *huh?* in a position close to instinctive cries [7], [42]. This would be one explanation for its similarity: it is innate, therefore all humans share it, therefore it assumes roughly the same form in all languages. This view is as hard to support as it is to discount, but we note four doubts.

#### No known phylogenetic precursor

Whereas laughter and pain cries (and by extension the conventionalized interjections associated with them) have demonstrable phylogenetic precursors in other mammals [43]–[46], there is, to the best of our knowledge, no evidence for an animal precursor of *huh?*. Nor is it obvious what the function and biological survival value of this precursor would be in primates which lack the kind of shared intentionality that underlies human cooperative communication [47], [48].

#### Not an involuntary response

Grunts and other non-linguistic vocalisations such as sneezes and pain cries are typically direct, involuntary responses to stimuli [49]. In contrast, the OIR interjection is selected for a specific purpose at a specific juncture in conversation from a larger system of alternative formats for initiating repair [15], [50]. A greater degree of agency over utterance and selection is characteristic of linguistic rather than instinctive expressions.

#### Acquired like a normal word

Whereas non-linguistic vocalisations like sneezes, cries and smiles are present at birth or develop soon after [51], [52], the acquisition of *huh?* follows a trajectory that is very similar to that of other linguistic items. In American English-speaking children, it is employed and responded to somewhat erratically at 2.5 years but perfectly at 5 years [37]. Related to this, the variability of laughs and screams appears to be much greater than what we find for *huh?*, and is not as strongly regimented by language [44], [53]–[55].

#### Parsimony

In terms of evolutionary history, language is a recent arrival that shows clear signs of being a bio-cultural hybrid: a complex adaptive system in continuous cultural evolution within a landscape of cognitive, cultural, and communicative factors [56]–[59]. Although some of our linguistic abilities are no doubt underpinned by genetic infrastructure, positing innateness for specific linguistic items would hardly be realistic given the timescale involved. Strong cultural universals do not necessarily imply strong innate biases [60] and strong innate biases are unlikely to evolve in cultural systems [61]. If there is a mechanism that can explain cross-linguistic similarity on a more proximate timescale, without resorting to genetic encoding, this is preferred on scientific principles of parsimony.

#### Convergence

A more plausible mechanism for the cross-linguistic similarity of *huh?* is convergent cultural evolution. This proposal sees *huh?* not as an arbitrary grunt but as a product of cultural evolution in the adaptive context of its interactional environment. The basic principle is well-known from biology: similar environmental constraints have led to the independent evolution of similar body plans in sharks and dolphins, and in the placental mammals of North-America and the marsupials of Australia. Likewise, we propose that the similarity of *huh?* in unrelated and distantly related languages is due to the fact that it is found in a strongly similar environment in all these languages. What is this environment like?

Conversations are built out of sequences of communicative moves between speakers [62], [63]. These moves –or ‘turns at talk’– are allocated in systematic ways and bear special sequential relations to each other [64], [65]. For instance, a question sets up an expectation that the addressee will provide a fitted response –in this case an answer– in the next move. Speakers inspect moves for their fittedness and aim to minimize gaps and overlaps between them. Speaker change most often takes only between 100–300 milliseconds, and deviations from the timing target can be treated as problematic [66], [67]. In order for this tight timing to work, planning a next turn often has to start well before the end of the preceding turn [68], [69]. Trouble in hearing or understanding is a regular feature of conversation [5], [47]. In the case of such trouble, planning and producing a fitted and timely response will be harder (indeed at times impossible), but the pressure to produce one will be just as strong. Given these pressures of turn-taking and formulation in conversation, a signal that indicates trouble should be minimal and easy to deploy. At the same time, given the communicative importance of indicating trouble (which if not solved might derail the conversation), such a signal should also clearly indicate a knowledge deficit and push for a response. These requirements are met rather precisely in the combination of minimal effort and questioning prosody that characterises the OIR interjection across languages.

#### Minimal effort

Many of the formal aspects of the OIR interjection minimize articulatory effort. The codaless monosyllable is the least marked syllable type across languages [68], [70]. The glottal onset, where present, is simply some constriction at the narrowest place in the vocal tract, and the unrounded low front central vowel is close to the neutral state of the articulators – both requiring minimal encoding, planning, and articulation [34]. Additionally, for Spanish phonetic corpus studies show that the vowel target of the interjection is the most frequently attested vowel [71], making retrieval, planning, and production easier [72]. These features render the OIR interjection well-fitted to the interactional environment of other-initiated repair. For the person initiating repair, the OIR interjection is quickly deployable from intention to articulation [68], and therefore easy to produce even under conditions of cognitive duress. For the addressee, the minimal form is a word that is unlike most content words and therefore –by Darwin's principle of antithesis [45]– a good signal that the other has no contentful response on offer.

#### Questioning prosody

If ‘minimal’ were the only design requirement, the most low-effort form possible would be enough. But to carry out the work of initiating repair, the OIR interjection also has to signal a knowledge deficit and indicate that a response is needed. We have seen that the intonation of the interjection appears to be calibrated to local systems of questioning prosody. In many languages this means that it has rising intonation – a contour that requires more effort than falling intonation [73], and (in English-speaking infants) has been shown to elicit greater attention [74]. In Cha'palaa and Icelandic, where the OIR interjection has falling intonation, it has a low central unrounded vowel – the vowel that is inherently most sonorous and acoustically salient due to the wide open oral cavity [75]. We propose that the questioning prosody and the acoustic salience of the interjection render it more adaptive for the function of OIR. As a question word devoid of semantic content, it expediently returns the floor to the original speaker and signals that there is trouble to be fixed.

In effect, *huh?* is an easy to produce, maximally underspecified question word – a tight fit of form and function found in language after language. We propose that this is the result of convergent cultural evolution: the interactional environment of other-initiated repair, present in every language investigated so far, provides a set of selective pressures that pull the interjection towards a similar form and that keep regular processes of language change from affecting the item. This process of convergent evolution explains the narrow bandwidth of the variation, but also the language-specific calibration of the items. To minimize articulatory effort, the OIR interjections of different languages will end up in the same low-effort area of the phonetic possibility space; yet to be recognised as questioning expressions, they will be calibrated to local phonological and prosodic subsystems.

We use ‘convergent evolution’ as a general term for the independent evolution of similarities in form and function. When ancestral forms are known, a distinction can be made between form/function convergence in species that are closely related (‘parallel evolution’) versus in species that are not closely related (‘convergent evolution’). However, this distinction is not always consistently made in biology and recently there have been proposals to use ‘convergent evolution’ as a general term [76]. We use the term in this general sense. Our proposal accounts for the present-day cross-linguistic similarity of *huh?*, but has to remain agnostic as to its ultimate origins – in the absence of historical language data it is impossible to tell whether the present-day forms go back to one ancestral form (a stabilising evolution scenario [77]) or whether they arose independently in different languages (an independent convergent evolution scenario [78]). In either case, the selective pressures are the same.

The convergent evolution proposal explains the forms documented so far, but also generates the prediction that in undescribed languages as well as newly emerging ones, we can expect to find a similar repair initiator that minimizes articulatory effort while making use of questioning prosody. Independently emerged sign languages of the deaf, though in a different expressive modality (visual-only instead of audio-visual), provide a good test case. Consistent with our proposal, in Argentinian Sign Language, repair can be initiated with a minimal sign that involves a raising of the eyebrows, the semiotic equivalent of questioning prosody [14],[79].

#### Conversational infrastructure and convergence of linguistic form

Apart from its explanatory and predictive value, the convergent evolution proposal offers a more general mechanism. For most words in most languages, there is no necessary connection between form and function. This is why words can change over time, and why we expect even words with similar functions to have different forms in unrelated languages. Accordingly, cultural evolutionary models of language change have tended to depict languages as collections of words evolving in utterances [80], [81], with various social and cognitive biases influencing transmission [82], [83] and with frequency of use as a primary factor influencing rates of change and divergence [84], [85]. However, our study points to a factor that may constrain divergence or diachronic drift: the selective pressures of specific conversational environments, which may cause convergent cultural evolution.

The possibility should not be surprising. After all, words evolve in utterances in conversation, so conversational infrastructure is part of the evolutionary landscape for words. We are referring here to the sequential infrastructure that serves as the common vehicle for language use – an infrastructure that may well predate more complex forms of language and that seems largely independent of sometimes radical differences between individual languages [63], [66], [86], [87]. A clear effect of this conversational ecology on the cultural evolution of linguistic items has not, to our knowledge, been observed before.

Though we have focused on *huh?* as a case study, the mechanism we propose has wider relevance. In our corpora, we have noted other items that are strongly similar in form and function across unrelated languages: continuers like *mm*/*m-hm* [88], hesitation markers like *uh/um* [89], [90], and change of state tokens like *oh/ah* [91]. It would be neither plausible nor parsimonious to propose that all of these have precursors in distinct innate grunts. Instead, we observe that these interjections all serve important discourse regulatory functions, and we propose that the reason they are so similar across languages is that common communicative needs and conversational infrastructure conspire to create, for each of them, a set of similar selective pressures constraining their evolution. The ultimate fit to the tight constraints of their conversational environments, these words stay put and help us conduct conversation in optimal ways. The approach followed in this study can be systematically extended to the larger set of discourse regulatory expressions and beyond, to explore further effects of conversational ecologies on language structure.

## Conclusions

We have presented evidence and arguments that *huh?*, or more precisely a short questioning interjection with the function of other-initiation of repair, is a universal word likely to be attested in similar form in all natural spoken languages. The similarity of this interjection across languages is unlikely to be specified in our genetic makeup and we argue that it is the result of convergent cultural evolution: a monosyllable with questioning prosody and all articulators in near-neutral position is the optimal fit to the sequential environment of other-initiated repair.

Our proposal invites closer attention to the infrastructure for social interaction that underlies language in use, and its possible influence on language structure. It also enhances existing models of language evolution and change by providing a mechanism for the convergent cultural evolution of linguistic items: conversational environments may exert selective pressure towards the evolution of common optimised forms, calibrated to local linguistic systems. Hence, we see how the investigation of a seemingly banal everyday word –previously characterised as a grunt or dismissed as a non-lexical sound– can shed light on the emergence and motivation of linguistic signs.

## Supporting Information

File S1 **Combined Supporting Information containing a description of the auditory analysis, the coding scheme used, and further information related to nasality and mouth aperture.**
(PDF)
