# Correction: Is "Huh?" a Universal Word? Conversational Infrastructure and the Convergent Evolution of Linguistic Items

**DOI:** 10.1371/journal.pone.0094620

**Published:** 2014-04-02

**Authors:** The PLOS ONE Staff

**Notice of Republication**

There were numerous errors throughout the article introduced during production, regarding the linguistic symbols. The article was republished on March 17, 2014. Please download the article again to view the correct version. The publisher apologizes for these errors.

## Supporting Information

File S1 **Originally published, uncorrected article.**
(PDF)

File S2 **Republished corrected article.**
(PDF)

## References

[1] Dingemanse M, Torreira F, Enfield NJ (2013) Is “Huh?” a Universal Word? Conversational Infrastructure and the Convergent Evolution of Linguistic Items. PLoS ONE 8(11): e78273 doi:10.1371/journal.pone.0078273 24260108 10.1371/journal.pone.0078273 PMC3832628

