# Supplementary material for: Is “Huh?” a Universal Word? Conversational Infrastructure and the Convergent Evolution of Linguistic Items
Source: PLoS One. 2013 Nov 8;8(11):e78273. doi: 10.1371/journal.pone.0078273 (PMC3832628; doi:10.1371/journal.pone.0078273)
Supplement: File S1 — Combined Supporting Information containing a description of the auditory analysis, the coding scheme used, and further information related to nasality and mouth aperture. (PDF) [file pone.0078273.s001.pdf]

Supporting information

1 Protocol for auditory analysis

In the auditory analysis described in the main text, all 196 interjection tokens were annotated independently by three analysts. Tokens were presented one by one in random order via a Praat script. The annotators listened to the audio clips of the extracted interjection tokens, which were accompanied by spectrograms and pitch tracks. No separate information about the language or recording was provided. The annotators independently coded all interjection tokens for five phonetic dimensions: pitch, mouth closure, onset, nasality, and vowel quality. A standardized set of values was used for every dimension. For vowels and consonants, coding allowed for observations outside the range. Table A illustrates the values available for coding in each phonetic dimension.

In the coding underlying the auditory analysis, the annotators used the following guidelines. Pitch was coded relative to the beginning of the item (i.e. is the end of the form higher (Rising), lower (Falling) or the same (Level)?). Mouth closure was coded as closed, open, or intermediate. Onset was coded as either zero (i.e. no consonantal onset), aspiration, glottal stop, or other. Nasalization was coded as either nasalized or oral. Aspiration and nasalized values were assigned regardless of their perceptual magnitude (see §4 below). Vowel quality was coded in four bins in a two-by-two grid representing frontness and height: front-low (E), mid-low (a), front-mid (e), and mid-mid (@). Vowels that feature values 'high' or 'back' were coded as other (o) — see §5 below.

Dimension	Categories	Value	Guidelines
**Pitch**	rising<br>falling<br>level	+1<br>-1<br>0	Relative to the beginning of the form, is the end of the form higher (rising), lower (falling), or the same (level)?
**Aperture**	closed<br>open<br>intermediate	-2<br>0<br>-1	Is the mouth closed, open, or intermediate between the two?
**Onset**	zero<br>aspiration<br>glottal stop<br>other	0<br>+1<br>-1<br>n.a.	If you hear any consonantal onset, code it as *h*, *g*, or *o*; if you don't hear any, code zero.
**Nasalization**	nasalized<br>oral	+1<br>0	If you hear any nasalization, code *n*, also if mouth is closed, code *n*.
**Vowel**	front mid<br>near-open front unrounded<br>mid central (schwa)<br>open central<br>other	+1,+1<br>0,+1<br>+1,0<br>0,0<br>0	The four vowel bins are fairly large. If mouth is closed, code *x* for "can't tell". 'Other' means any value in 'high' or 'back' areas.
**All**	can't tell	exclude	In case of overlap, low volume, bad audio quality, or other causes for inaudibility.

Table A. Coding categories, guidelines, and values used in computing the graded measures. A value of *n.a.* means that this value did not occur in the coding. A value of *exclude* means that tokens coded as "can't tell" in a given dimension are excluded from consideration in that dimension.

In order to analyse these judgements, we combined the annotations into cumulative graded measures using the values in Table A. These measures were computed as follows, taking the Intonation category as an example. Annotators coded for three basic intonation contours: Rising (r), Level (l), and Falling (f). Every coding judgment was counted as a single vote upwards (for Rising), downwards (for Falling) or neutral (for Level). Three judgements of a token as Rising would thus amount to a value of +3. Two judgement of a token as Falling and one as Level would amount to a value of -2. Variance in judgments was allowed because phonetic realisations are always gradient and partly observer-dependent.

The combined scores allow us to visually present the gradient variation in product plots [1]. In product plots, the area of squares is propertional to token count: larger squares mean more tokens. Thus Figure 5 in the main text shows that most tokens in most languages have rising intonation, virtually none have level intonation, and the tokens of two languages (Cha'palaa and Icelandic) are falling. Similarly, Figure A below shows that most tokens in most languages show at least some degree of nasalisation. The procedure of independent coding and subsequent summation gives us graded analytic judgements that are better empirically grounded than simple descriptions in an arbitrary symbol system (e.g. the International Phonetic Alphabet).

2 Audio files

The 196 audio files are available upon request to the first author.

3 Intonation

Figure 6 in the paper displays pitch tracks for interjections in Spanish (mostly rising) and Cha'palaa (mostly falling). It shows that four of the Spanish contours did not have a complete final rise. In these cases the contours rise and then have a short fall to a mid tone. We inspected these contours and found that the final fall occurred in final parts of the signal with very weak intensity. This final fall is not very salient, and one may wonder whether the Spanish speakers targeted a final mid tone in these cases, as the figure might suggest, or whether they instead targeted and achieved an audible high boundary tone, and then inadvertently produced a mid tone as their glottis adopted their default non-speech configuration during the last portion of the interjections. Some Cha'palaa contours displayed a rather flat pattern. Inspection of these examples revealed that their final portions presented creaky voice contributing to the auditory impression of a low boundary tone. Pitch could not be measured in these portions, which explains why they appear as truncated flat contours in normalized time.

4 Onset

Figure 7 in the paper displays onsets by language. The guidelines for coding focused on any hearable onset, and so the auditory judgements displayed in this figure do not provide information on the strength of articulation of the onset. However, we found that the interjection tokens differed quite markedly in this regard. Many Icelandic tokens have a strong breathy /h/ (e.g. Icelandic_01, Icelandic_13, Icelandic_14, Icelandic_18), whereas the Spanish tokens that were marked as having this same sound were much more subtle (e.g. Spanish_08, Spanish_15). The wide distribution of

Spanish tokens over the space reflects the fact that the Spanish data consists of lab quality recordings [2] in which even slight glottal constriction or frication can be detected easily.

5 Nasality and mouth aperture

Some degree of nasality was perceived in the great majority of cases (Figure A).

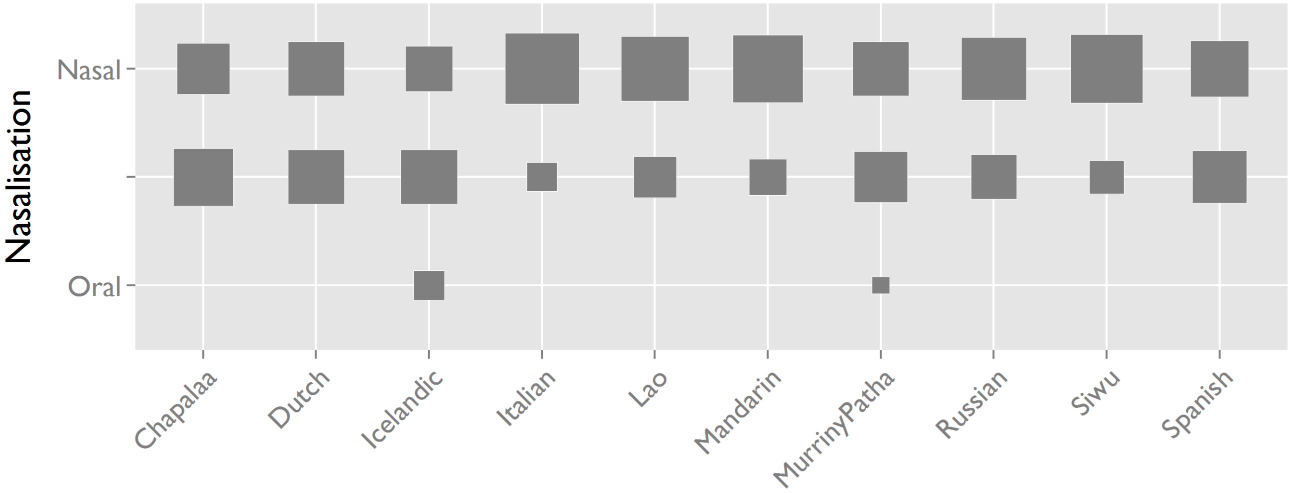

Figure A. Nasality by language. Across languages, most interjection tokens have at least some nasality. This is likely connected to the fact that a slightly lowered velum is the default position for the articulators.

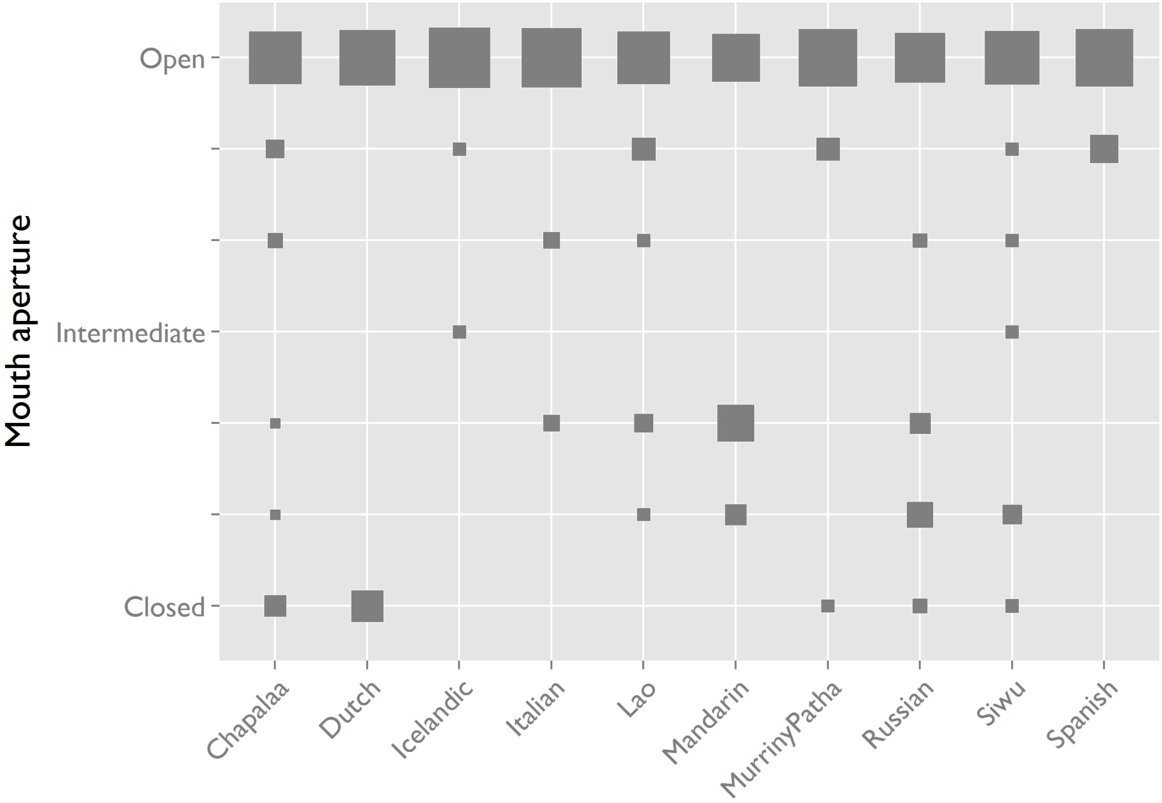

Figure B. Mouth aperture by language. The majority of interjection tokens are produced with an open mouth. Many languages allow underarticulated closed-mouth variants of the interjection (e.g. *m?*), but this is not the most common form in any language.

6 “Other” values

The coding protocol for vowels and consonants explicitly allowed observations outside the range found in preliminary observations. However, this possibility was used for only 3 out of 196 tokens: Chapalaa_21 (described by one coder as “other: bit higher and bit more central”, but as /a/ by the other two coders), Siwu_18 (described by one coder as “other: very back from a” but as /a/ by the others), and Dutch_06 (a token in overlap with other speech, described as “mid central” and “can’t tell” by the others).

7 References

1. Wickham H, Hofmann H (2011) Product plots. Visualization and Computer Graphics, IEEE Transactions on 17: 2223–2230.

2. Torreira F, Ernestus M (2010) The Nijmegen corpus of casual Spanish. Proceedings of the Seventh conference on International Language Resources and Evaluation (LREC’10), Valletta, Malta. European Language Resources Association (ELRA). p. 2981.
